# Electroencephalography (EEG)-Based Neural Emotional Response to Flower Arrangements (FAs) on Normal Elderly (NE) and Cognitively Impaired Elderly (CIE)

**DOI:** 10.3390/ijerph19073971

**Published:** 2022-03-26

**Authors:** Juan Du, Jiali Yin, Xiaomei Chen, Ahmad Hassan, Erkang Fu, Xi Li

**Affiliations:** College of Landscape Architecture, Sichuan Agricultural University, Chengdu 611130, China; dujuana@sicau.edu.cn (J.D.); yinjl2022@163.com (J.Y.); m18307038977@163.com (X.C.); ahmad.hassan@sicau.edu.cn (A.H.); erkang@sicau.edu.cn (E.F.)

**Keywords:** EEG, flower arrangement, elderly, horticultural therapy, neural emotion

## Abstract

Background: The purpose of this study is to explore the differences and similarities of EEG -based neural emotional response toward flower arrangements (FAs) between the normal elderly (NE) and cognitively impaired elderly (CIE) in arranging flowers. Methods: The study participants included 16 elderly individuals: eight elderly people with normal cognitive function and eight elderly people with cognitive dysfunction. They were divided into two groups to arrange flowers, and six mood indicators (Engagement, Excitation, Focus, Interest, Relaxation and Stress) were measured with EEG before and after the experiment. Results: The similarities were that there was no significant difference in Excitement, Relaxation and Stress between pre-test and post-test for NE and CIE. The differences were that there was a significant difference on Engagement and Interest in CIE, and they both increased, but there was no difference with respect to them in NE. While there was a significant difference on the Focus of NE, it was decreased, but there was no difference for it with respect to CIE. Conclusions: A similarity on EEG-Based Neural Emotional Responses to flower arrangements between NE and CIE was that they both felt relaxation. The differences were that the Focus of NE decreased and the Interest and Engagement of CIE increased. CIEs were more interested and engaged in FAs.

## 1. Introduction

### 1.1. The Effects of HT on Elderly Psychophysiology

China currently has the largest number of elderly people in the world [1]. Since 2013, China has entered an aging society [2]. In 2019, the proportion of 65-year-old individuals in China was 12.6 percent [2]. The number of senior citizens in China will exceed 400 million by 2050 [3]. With the increase in aging, the number of elderly people with dementia is increasing rapidly. By 2030, there will be 18.79 million people over 60 years old with dementia in China, which is about double the number in 2010 [4]. Countries facing an aging population must strive to maintain the physical and mental health of older persons in order to reduce the economic burden of medical resource consumption in this age group. 

Horticultural therapy refers to an effective method to adjust and renew people who need to improve their body and spirit by using plant cultivation and gardening activities in social, educational, psychological and physical aspects [5]. Gardening activities might enhance physiological and physiological relaxation in older adults [5,6]. Gardening improves the arthritis of the elderly, and it is beneficial to the control of blood pressure and diet [7,8]. It can effectively eliminate the interference of old people’s anxiety and annoyance and depression in addition to other bad emotions [6,8,9,10,11]; promote the physical and mental health of the elderly; and improve the quality of life of the elderly. Gardening promotes hand strength, pinch force and overall physical health [12]. Horticultural program improved attitudes toward aging, hand eye coordination and the sense of hope in older participants [13]. The Profile of Mood States scores of the elderly indicated significant improvements for Rocky Leaf Prints and Herb Tasting and Smelling [14]. One study demonstrated that a short peony-viewing program, especially at the full bloom stage with completely opened and large tree peony flowers, would be a promising therapeutic method for improving physiological functions as well as an effective psychological relaxation strategy for middle-aged and elderly individuals [15]. An experience sampling pilot study among older adults living in a nursing home was conducted, and the results show that participants levels of the cognitive and motivational variables increased during horticultural activities [16]. Another study demonstrated the potential ability of horticultural therapy to improve the stress levels and physical functional abilities of elderly people with mental health problems [17]. HT, in reducing plasma IL-6, may prevent inflammatory disorders and, by maintaining plasma CXCL12 (SDF-1α), may maintain hematopoietic support for the brain for older people [8]. Currently, many studies on HT show that HT can effectively improve the psychological and physiological indicators of the elderly.

Elderly individuals with dementia have a series of cognitive disorders with respect to memory, language, thinking and judgment ability, which may be accompanied by agitation and other behavioral psychiatric symptoms. Promoting the elderly with dementia to participate in daily life in the nursing unit, to carry out interesting and meaningful activities and to increase communication and social contact is considered to be a very effective intervention [18]. Horticultural therapy (HT), as an important means of prevention, treatment and rehabilitation of dementia, has attracted increasing attention from scholars at home and abroad [19]. For the specific positive effects of gardening on patients with Alzheimer’s disease, refer to D Andrea et al. [20], who found evidence of it promoting creativity, self-esteem, social interaction, sensory stimulation, gross and fine motor skills and hand-eye coordination. Thaneshwari et al. [21] recommended that therapeutic gardens be designed specifically for the care of certain types of patients, such as Alzheimer gardens. An innovative life review program combined with horticultural activities on Chinese seniors with probable dementia showed a significant positive effect on participants’ cognitive ability and meaning of life [22]. A quantitative review by Zhao et al. [23] on the benefits of gardening on people with dementia reported improvements in cognitive function, agitation, emotional state and engagement. The 24-session gardening program significantly improved cognitive function and brain-derived neurotrophic factor levels related to cognitive health factors of the participating elderly individuals [24]. Additionally, there are examples of HT studies in elderly people with dementia: One course of gardening (four weeks, once a week) can effectively improve the mental and physical health of the elderly with dementia [19]; therapeutic gardens should be used to improve the health and wellbeing of dementia patients [25]; and HT can improve depression and agitation in dementia patients [26,27,28,29]. Recent reviews and studies [25,30,31] found that including therapeutic gardens in care environments has positive effects on agitation, behavior, walking, stress levels, self-esteem, depression and aggressiveness.

### 1.2. The Study of the Use of EEG

Electroencephalography (EEG), which records electrical activity inside the brain, is commonly used in research and clinical settings. It has been widely used to reflect the neural emotions of the human brain [32,33,34,35], and different neural emotional indicators (engagement, excitation, focus, meditation, frustration, interest, relaxation and stress) can be output through an EEG device [36,37]. Because emotional indicators are relatively objective compared with nonphysical cues, EEG is reliable for emotional recognition [38]. In this study, we used the EEG headset to collect brainwave signals, and six emotional indicators were outputted (Engagement, Excitement, Focus, Interest, Relaxation and Stress). This research refers to a previous study [37] on an EEG headset, Emotiv’s explanation, to present the working definitions of each emotional parameter: “Engagement” reflects the degree of immersion, investment or attraction; “Excitement” represents the level of arousal of the individual and is associated with a high degree of arousal; “Focus” is a high state of arousal, reflecting high attention; “Interest” indicates the degree of liking or dislike to the current environment, which represents the attractiveness of the environment; “Relaxation” is associated with low levels of arousal and when people are in a relaxed state, their heart rate slows, their blood pressure drops and their level of arousal decreases. “Stress” refers to the psychological tension reaction or state formed under the action of individual anxiety or fear, which represents the negative valence dimension of people’s emotions such as frustration and disappointment.

Based on previous studies on horticultural therapy for the elderly, no one has studied the differences and similarities of EEG-based neural emotional response to horticultural activity between normal elderly and cognitively impaired elderly. We hypothesized that the neuro-emotional responses to flower arrangements would be different between the two groups. This study explores the differences and similarities with respect to neuroemotional responses to flower arrangements of the two groups of elderly, and it provides references for different elderly groups to design gardening activity.

## 2. Materials and Methods

### 2.1. Participants

Participants came from a nursing home in Wenjiang District, Chengdu, Sichuan Province, China. In this experiment, 16 volunteer aged 85 ± 8.7 years (mean ± SD) joined this study, including 8 elderly individuals with normal cognitive function and 8 elderly individuals with cognitive dysfunction. They were divided into a normal elderly group and cognitively impaired group. All old people participating in the experiment signed an informed consent form for the experiment, and the experiments were conducted with the approval of the local ethics committee of the College of Sichuan Agricultural University, China.

This experiment was carried out in locations of institutional care in Chengdu, China, on 19–22 April 2021, 9:00–11:30 a.m. and 14:00–17:00 p.m. The room was 6 m long and 3.6 m wide, and it was kept quiet during the experiment. Indoor temperature, humidity and illumination were maintained at 22–24 °C, 50–55% and 500 lux, respectively.

### 2.2. Materials

**Figure 1:**
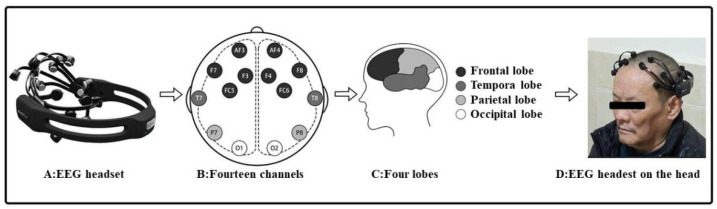
EEG headset.

All flower materials, scissors and vases were purchased from a florist. We used an EEG headset (Figure 1) to collect brainwave signals. The device is nonintrusive and has the advantage of multichannel acquisition. Its 14 electrodes (AF3, AF4, F3, F4, F7, F8, FC5, FC6, T7, T8, P7, P8, O1 and O2) cover four brain lobes (frontal cortex, temporal cortex, parietal cortex and occipital cortex). Numerous studies have confirmed the precision performance of the instrument [39,40,41]. EEG data collected by the electrodes were sent to a computer hard disk by using Bluetooth. EEG activity was then analyzed, and performance metric data containing six emotional indicators were outputted (Engagement, Excitement, Focus, Interest, Relaxation and Stress). The device outputs six mood readings per minute, and data were recorded at one-minute intervals during the experiment.

### 2.3. Procedure

**Figure 2:**
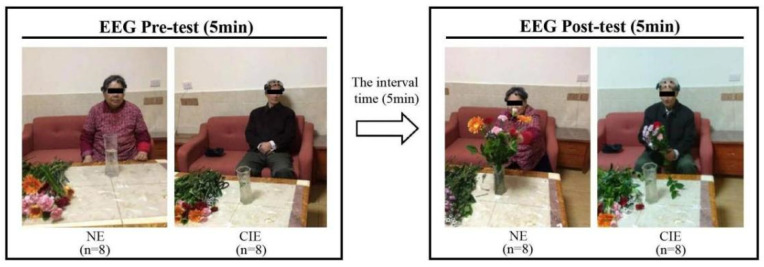
Study procedure (EEG pre-test: test neural parameters before FA. EEG post-test: test neural parameters during FA).

The researcher will demonstrate in advance how to make a flower arrangement until the groups fully understand it. The subjects selected flowers, trimmed them and inserted them into a vase with water. Then, the position of the flower branches was adjust appropriately until they are satisfied, and the flower arrangement is then complete. The entire process was as follows: At first, subjects would have a rest in a seating position for 5 min to keep their brain calm, and then they would put on the EEG device. This study analyzed 14 channels in four brain lobe regions: F3 and F4 in the frontal lobe, which controls cognition, language, emotion and behavior; T7 and T8 in the temporal lobe, which controls audio-visual memory and language processing; P7 and P8 in the parietal lobe, which controls sensory connection and reception; and O1 and O2 in the occipital lobe, which controls all visual information. EEG activity would be continuously measured during the experiment. The date was recorded one time at one-minute intervals, and it was captured 5 times before the flower arrangement (pre-test). Then, data would be collected 5 times again during the period in which they began to arrange flowers for 10–15 min (post-test). We took the average of five times as the test value. The interval between the pre-test and post-test is five minutes (Figure 2).

### 2.4. Statistical Analysis

Statistical analysis was carried out by SPSS 24.0 (SPSS, Armonk, NY, USA). One-way ANOVA and the Wilcoxon signed-rank test were used to analyze the mean values of pro-test data and post-test data.

## 3. Results

**Figure 3:**
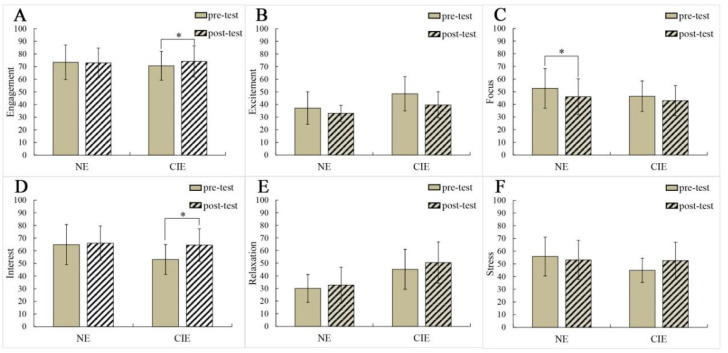
Significance analysis of the pro-test and post-test data (*n* = 8; mean ± standard error; * *p* < 0.05). ((**A**) Engagement (**B**) Excitement (**C**) Focus (**D**) Interest (**E**) Relaxation (**F**) Stress).

In the process of data analysis, it was found that Engagement and Excitement of NE and Engagement, Relaxation and Stress of CIE did not meet the conditions of the parameter test; thus, the Wilcoxon signed-rank test was adopted. The other neuroemotional indicators can be analyzed by one-way ANOVA. The significant differences of six neuroemotional parameters before and after the experiment were analyzed. There was no significant difference on the indicator of Excitement (Figure 3B), Relaxation (Figure 3E) and Stress (Figure 3F) of the two groups, but there was a significant difference on the Focus of NE (F = 5.895, *p* < 0.05) (Figure 3C). This showed that the NE’ attention decreased during arranging flowers, while according to the results of the Wilcoxon signed-rank test, there was a significant difference on the Engagement of CIE (Figure 3A). This showed a significant increase in the participation of CIE during flower arrangements. In addition, as shown in Figure 3D, there was a significant difference in the Interest of CIE (F = 10.230, *p* < 0.01). This suggests that CIE showed a significant increase in interest when arranging flowers. The significant increase in these indicators indicates that CIE liked to arrange flower.

The correlation analyses of various brainwave neuroemotional indicators of NE showed (Table 1) that Focus and Excitement (r = 0.27, *p* < 0.05), Interest and Relaxation (r = 0.71, *p* < 0.01) and Focus and Stress (r = 0.52, *p* < 0.01) were significantly positively correlated, respectively. However, Focus and Relaxation had significant negative correlations (r = −0.37, *p* <0.01).

The correlation analyses of various brainwave neuroemotional indicators of CIE showed (Table 2) that Focus and Excitement (r = 0.55, *p* < 0.01), Interest and Engagement (r = 0.62, *p* < 0.01), Interest and Excitement (r = 0.51, *p* < 0.01), Interest and Focus (r = 0.67, *p* < 0.01), Relaxation and Excitement (r = 0.67, *p* < 0.01), Relaxation and Interest (r = 0.51, *p* < 0.05), Stress and Engagement (r = 0.53, *p* < 0.01), Stress and Focus (r = 0.77, *p* < 0.01), Stress and Interest (r = 0.82, *p* < 0.01) and Stress and Relaxation (r = 0.47, *p* < 0.05) had significant positive correlations, respectively.

## 4. Discussion

This study presented differences and similarities on (EEG)-based neural emotional responses to flower arrangements between NE and CIE. The similarities were that there was no significant difference in Excitement (Figure 3B), Relaxation (Figure 3E) and Stress (Figure 3F) between pre-test and post-test for NE and CIE. The differences were that there was a significant difference in Engagement (Figure 3A) and Interest (Figure 3D) of CIE; they both increased. However, there were no differences in them for NE. While there was a significant difference in Focus (Figure 3C) of NE, it decreased, but there were no differences for it in CIE.

Although there was no significant difference in the Relaxation value between pro-test and the post-test, as observed in Table 1, the Focus of NE was negatively correlated with Relaxation: Focus decreased significantly (Figure 3C), indicating that Relaxation increased, as observed from Figure 3E. For CIE, their Interest was positively correlated with Relaxation (Table 2): Interest increased (Figure 3D), indicating that Relaxation increased too. Therefore, we can obtain one conclusion whereby FA made NE and CIE feel relaxed. It has a positive effect on the neuro-emotion of participants. These findings are broadly consistent with other experiments that examined psychophysiological responses of older adults with respect to gardening activity [5,19,25,28,41]. On the one hand, in the HT study on NE, Hayashi [42] reported that HT, which allows for engagement with nature, has been suggested to mitigate stress and improve mood states. Hui [43] showed that HT can enhance the mental health, cognitive functioning and physical health of NE. Hassan [6,44] thought horticultural activity might enhance physiological and psychological relaxation in older adults. On the other hand, in the HT study on CIE, Marianne [45] had noted in a review on the benefits of horticultural therapy in the care of dementia patients that these types of nonpharmacological interventions may improve wellbeing and affect and reduce the occurrence of disruptive behavior. Patrick [25] had suggested that therapy gardens should be used to improve the health and wellbeing of Alzheimer’s disease (AD) and dementia patients. These findings confirm the efficacy of HT in CIE; in this study, FA also promoted neuro-emotional relaxation in CIE.

However, there was a significant reduction in the Focus of NE (Figure 3C), while there was no difference in it for CIE. We know one important theory, namely, Attention Restoration Theory (ART) [46,47,48]. In green spaces, the walking group had lower “Focus” than the sitting group [36]. The walking group’s directional attention was reduced. Kaplan [46] suggested the theory that people’s ability to suppress distractions will reduce with vegetation density, which naturally helps to supplement directed attention over time. Participants’ Focus increased with vegetation density and integrated sound environment [35]. In this experiment, NE would want to pursue the beauty of the works more, and they would repeatedly trim and arrange flower materials until they were satisfied. During this process, they constantly consumed directional attention, According to ART, their directed attention would decrease, which may be the reason for the decrease in Focus. At the same time, this study found that there was a significant increase in Engagement (Figure 3A) and Interest (Figure 3D) in CIE, but there was no difference in them for NE. This suggests that FA is more attractive for CIE, who were more engaged and immersed in it. As we all know, flower arranging is a very popular gardening activity. After making a real flower basket, forty Chinese women’s blood pressure and anxiety scores dropped significantly, and Alpha brainwaves increased significantly [49]. The increase in alpha waves indicates that the brain is in a pleasurable sate. This shows that FA can promote people’s physical and mental health. Seon-Ok Kim showed that the total mood disorder score of elderly was lowered after FA, indicating a positive effect on the mood of participants [50]. FA can effectively reduce the total emotional disorder and anxiety of the Psychology teacher [51]. These studies confirmed the health benefits of FA. Furthermore, the structured floral arrangement program encouraged continuous participation for cognitive intervention and was useful for ameliorating dysfunctions in visuospatial memory and recognition in patients with neurocognitive disorder [52]. This confirms the benefits of FA for CIE. More FA activities can be considered in horticultural therapy for CIE in the future.

There are two limitations in this study. First, the sample size of subjects is limited. In future studies, more subjects can be considered to participate in the experiment to obtain more reliable experimental data. Secondly, this experiment only studies the influence of gardening activities such as flower arrangement on two kinds of old people, and different gardening activities may have different effects on them. In the future, we can consider studying the influence of more types of gardening activities on the physical and mental health of the two kinds of old people, developing a more sophisticated program of horticultural therapy for the elderly.

## 5. Conclusions

This study explored the differences and similarities on (EEG)-Based Neural Emotional Response to flower arrangements between NE and CIE. The conclusions presented a similarity: both groups felt relaxation in FA. The differences were that the Focus of NE decreased and the Interest and Engagement of CIE increased. CIEs were more interested and engaged in FA. In the future, FA should be considered more in horticultural therapy for CIE. Of course, considering that flower arrangements are also beneficial for NE, they can also make flower arrangement a hobby in order to enrich their elderly life.

## Figures and Tables

**Table 1:** Correlation analysis of neuroemotional indicators of NE (*n* = 8).

Variable	Mean	SD	1 Engagement	2 Excitement	3 Focus	4 Interest	5 Relaxation
1 Engagement	78.30	11.91					
2 Excitement	66.73	10.92	−0.05				
3 Focus	31.83	19.47	−0.14	0.27 *			
4 Interest	49.15	12.74	0.11	0.16	−0.21		
5 Relaxation	31.60	13.86	0.16	−0.01	−0.37 **	0.71 **	
6 Stress	60.28	22.33	−0.04	−0.24	0.52 **	0.25	0.17

* *p* < 0.05, ** *p* < 0.01.

**Table 2:** Correlation analysis of neuroemotional indicators of CIE (*n* = 8).

Variable	Mean	SD	1 Engagement	2 Excitement	3 Focus	4 Interest	5 Relaxation
1 Engagement	71.00	16.12					
2 Excitement	61.43	13.65	−0.03				
3 Focus	37.78	17.17	0.38	0.55 **			
4 Interest	44.03	11.75	0.62 **	0.51 **	0.67 **		
5 Relaxation	42.35	18.19	−0.20	0.67 **	0.23	0.51 *	
6 Stress	48.70	26.88	0.53 **	0.37	0.77 **	0.82 **	0.47 *

* *p* < 0.05, ** *p* < 0.01.
